# The TSC1/2 Complex Controls *Drosophila* Pigmentation through TORC1-Dependent Regulation of Catecholamine Biosynthesis

**DOI:** 10.1371/journal.pone.0048720

**Published:** 2012-11-07

**Authors:** Diana Zitserman, Sapna Gupta, Warren D. Kruger, Magdalena Karbowniczek, Fabrice Roegiers

**Affiliations:** Fox Chase Cancer Center, Philadelphia, Pennsylvania, United States of America; Baylor University, United States of America

## Abstract

In *Drosophila*, the pattern of adult pigmentation is initiated during late pupal stages by the production of catecholamines DOPA and dopamine, which are converted to melanin. The pattern and degree of melanin deposition is controlled by the expression of genes such as *ebony* and *yellow* as well as by the enzymes involved in catecholamine biosynthesis. In this study, we show that the conserved TSC/TORC1 cell growth pathway controls catecholamine biosynthesis in *Drosophila* during pigmentation. We find that high levels of Rheb, an activator of the TORC1 complex, promote premature pigmentation in the mechanosensory bristles during pupal stages, and alter pigmentation in the cuticle of the adult fly. Disrupting either melanin synthesis by RNAi knockdown of melanogenic enzymes such as *tyrosine hydroxylase* (TH), or downregulating TORC1 activity by Raptor knockdown, suppresses the Rheb-dependent pigmentation phenotype in vivo. Increased Rheb activity drives pigmentation by increasing levels of TH in epidermal cells. Our findings indicate that control of pigmentation is linked to the cellular nutrient-sensing pathway by regulating levels of a critical enzyme in melanogenesis, providing further evidence that inappropriate activation of TORC1, a hallmark of the human tuberous sclerosis complex tumor syndrome disorder, can alter metabolic and differentiation pathways in unexpected ways.

## Introduction

**Figure 1:**
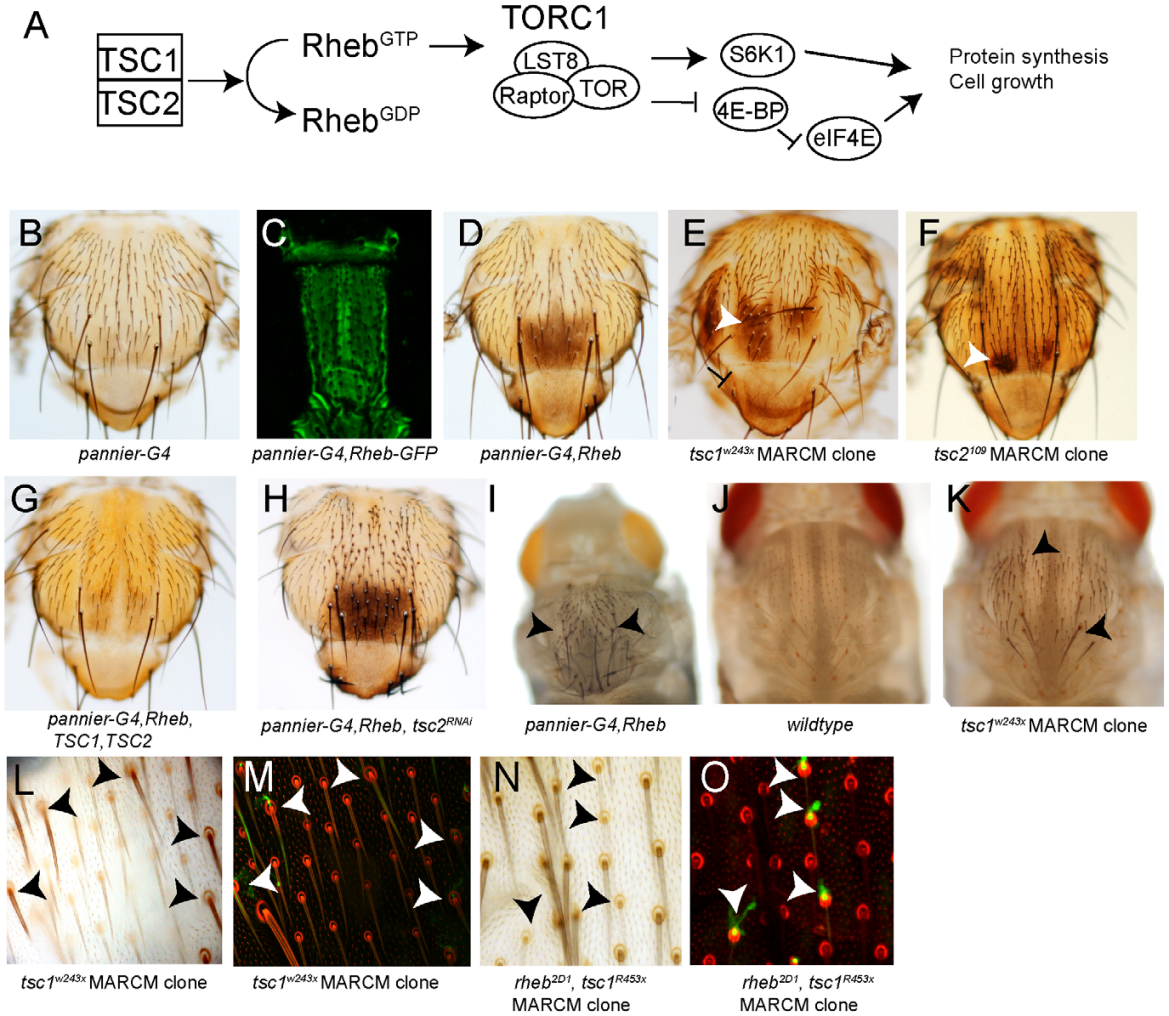
Rheb drives increased pigmentation of the pupal and adult cuticle. The evolutionarily conserved TSC pathway regulates protein synthesis and cell growth through activation of TOR complex 1 (TORC1) (A) [12]. Uniform pigmentation of the adult male thorax in *pannier-Gal4/+* (we will use the abbreviation “*-G4*” for Gal4 in this and subsequent figures) (B). Pattern of expression of *pannier-Gal4, UAS-Rheb-GFP* on the pupal thorax (C). “trident pattern” pigmentation in the posterior thorax *UAS-Rheb*, *pannier-Gal4* adult male fly (D). MARCM clones of *tsc1^w243x^* and *tsc2^109^* (E,F), exhibit posterior pigmentation (white arrowheads) in clones (clones marked with GFP, see L­O). *UAS-TSC1* and *UAS-TSC2* suppress the increased growth and pigmentation in *pannier-Gal4, UAS-Rheb* flies (G). *UAS-TSC2^RNAi^* enhances the increased growth and pigmentation in *pannier-Gal4, UAS-Rheb* flies (H). *pannier-Gal4, UAS-Rheb* shows premature bristle pigmentation in a dorsal stripe in stage P11 pupa (I). Pupa, stage P10 in wildtype (J) and *tsc1^w243x^* MARCM clones (K-M), GFP-marked (arrowheads) *tsc1^w243x^* bristles pigment prematurely, red in M and O is autofluorescence of the cuticle. Premature pupal bristle pigmentation is suppressed in *rheb^2D1^, tsc1^R453x^* clones, marked by arrowheads (N,O) and GFP (green, O). Genotypes of flies: *Y/w, UAS-dicer2; pannier-Gal4/+*(B), *Y/w, UAS-dicer2; UAS-Rheb-GFP/+*, *pannier-Gal4/+*(C), *Y/w, UAS-dicer2; UAS-Rheb/+*; *pannier-Gal4/+*(D,I), *w/yw, Ubx-flp; scabrous-Gal4,UAS-Pon-GFP, UAS-Tau-GFP/+; FRT82B, tsc1^w243x^/FRT82B tub-Gal80* (E, K–M), *w/yw, Ubx-flp; scabrous-Gal4,UAS-Pon-GFP, UAS-tau-GFP/+; tsc2^109^ FRT80B/tub-Gal80 FRT80B* (F). *Y/w; UAS-Rheb/+*, *pannier-Gal4/UAS-tsc1,UAS-tsc2* (G), *Y/w, UAS-dicer2; UAS-Rheb/+*, *pannier-Gal4/UAS-tsc2^RNAi^* (H). *w/yw, Ubx-flp*; *scabrous-Gal4,UAS-actin-GFP/+; FRT82B rheb^2D1^, tsc1^R453x^*/*FRT82B tub-Gal80* (N,O).

During development, organisms must coordinate growth, proliferation and differentiation. The TORC1 complex is an evolutionarily conserved central node in coordination of cell growth by driving protein synthesis in response to growth factor signals and the availability of amino acids [1]. At the cellular level, increased TORC1 activity results in increases in cell size, and in some cases, increased cell proliferation, as well as activation of stress response pathways [2]–[5] Regulation of TORC1 activity is mediated by the activity of Rheb GTPase. Rheb in turn is controlled by a heterodimeric complex composed of products of the *tuberous sclerosis complex 1* and *2* genes (TSC1 and TSC2, or hamartin and tuberin, respectively) which act together as a GTPase-activating protein (GAP) to limit Rheb by maintaining it in a GDP bound state (Fig. 1A). Chronic activation of the TORC1 complex is associated with human pathologies such as the Tuberous Sclerosis Complex, a tumor suppressor gene syndrome characterized by growth of benign tumors in multiple organs along with neurological manifestations resulting from inactivating mutations in either *TSC1* or *TSC2* genes [6]. During development, inappropriate TORC1 activity can affect the timing and fidelity of cell fate assignments [7], [8], but the mechanisms governing these defects are unclear. Here we show that chronic activation of TORC1 in the *Drosophila* pupal epidermis results in hyperpigmentation of mechanosensory bristles and adult cuticle due to increased levels of tyrosine hydroxylase.

## Results

### TSC1 and TSC2 Regulate *Drosophila* Adult Pigmentation Through Rheb

In a previous study, we showed that increased Rheb activity results in cell fate specification defects in the mechanosensory bristle lineage in *Drosophila,* consistent with inappropriate Notch activity [8]. Here, we sought to determine whether increased Rheb activity causes other differentiation defects during *Drosophila* pupal development that would be visible on the adult fly. We used the Gal4/UAS system [9] to drive high levels of Rheb expression in pupal epithelial tissues with *pannier-Gal4*. The resulting flies showed an increase in cell size and, at a low frequency, duplication of external cells in the mechanosensory organs. In addition, we noted the appearance of increased cuticular pigmentation in adult flies. The increased pigmentation pattern is particularly striking along the dorsal midline of the thorax and abdomen, where *pannier-Gal4* drives strong expression (Fig. 1A–D). In contrast to wildtype controls, Rheb overexpressing flies showed a dark patch of pigment in the central posterior region of the thorax, and a broadening of the dorsal pigment stripe in abdominal segments A3 and A4 (Fig. S1A–B). We noted that although Rheb was expressed throughout the *pannier-Gal4* expression domain (Fig. 1B), the darkening of the thoracic cuticle was almost exclusively confined to posterior-most region of the notum, a region referred to as the “trident”. In order to exclude the possibility that the cuticle darkening phenotype in this cross was due to a genetic background effect, we crossed *pannier-Gal4* to two independent P-element insertions of UAS-Rheb-GFP and to the *rheb^AV4^* allele, which contains a UAS-bearing P element insertion within the 5′ UTR of Rheb (Fig. S1C). In all cases, we observed darkening of the cuticle on both the thorax and abdomen in a similar pattern.

TSC2 functions as a GTPase activating protein (GAP) for Rheb, and along with its TSC1 binding partner, maintain Rheb in a GDP-bound, inactive state (Fig. 1A) [10]–[12]. In order to test whether the thoracic increased pigmentation phenotype is due to inappropriate activity of endogenous Rheb, we used the MARCM (mosaic analysis with a repressible cell marker) system [13] to generate clones of either mutant *tsc1* or *tsc2*. In adult flies of the appropriate genotypes, we observed regions of the thorax that contained ectopic pigmentation, as well as increased cell size, and duplicated bristles on the pupal thorax, however, the increased pigmentation and thickness of the adult cuticle precluded us from visualizing the GFP marker to positively identify these regions as *tsc1* or *tsc2* mutant (Fig. 1E, F). Nonetheless, consistent with the idea that TSC1/2-dependent regulation of Rheb contributes to control of pigmentation, overexpression of TSC1 and TSC2 strongly suppressed the pigmentation and growth phenotypes in Rheb overexpressing flies (Fig. 1G), while knockdown of *tsc2* by RNAi significantly enhanced the Rheb-induced pigmentation (Fig. 1H, and Fig. S1D) on the adult thorax.

Pigmentation of the pupal cuticle begins at the late stages of metamorphosis (stages P10) [14], proceeding as an anterior-posterior wave of mechanosensory bristle pigmentation, followed by post-eclosion cuticular tanning [15]. We therefore evaluated the onset of pigmentation in either Rheb overexpressing or *tsc1* mutant mechanosensory bristles. We saw that while the lateral thoracic mechanosensory bristles, which are outside the *pannier-Gal4* expression domain, were unpigmented in stage P10 *pannier-Gal4,* UAS-Rheb pupa, the dorsal bristles within the *pannier-Gal4* expression domain were strikingly dark along a broad dorsal stripe (Fig. 1I). Similarly, we found that in *tsc1* MARCM clones (which could be definitively identified by strong GFP expression at this developmental stage), mechanosensory bristle pigmentation initiated earlier than in neighboring wildtype bristles (Fig. 1J–M). In contrast, pigmentation was delayed compared to wildtype bristles in marked *rheb, tsc1* double mutant clones (Fig. 1N, O), suggesting that *rheb* is required for the precocious pigmentation in *tsc1* clones. Taken together, we conclude that Rheb activity is a limiting factor in the timing and degree of adult pigmentation on the thorax and abdomen.

### TORC1 Regulation of S6K and eIF4E is Required for Rheb-induced Pigmentation

**Figure 2:**
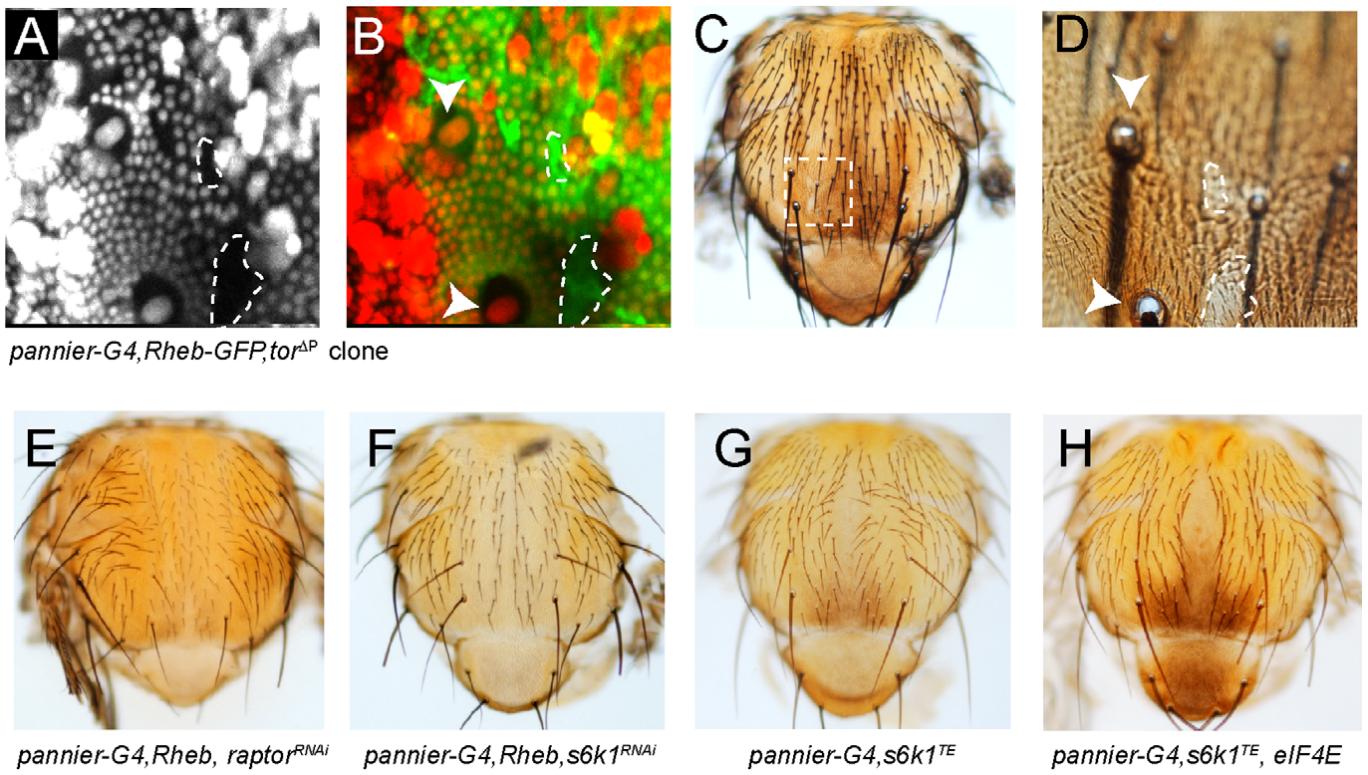
TORC1 and S6 kinase-dependent pigmentation of the adult cuticle. Pigmentation and bristle growth phenotype in *UAS-Rheb-GFP*, *pannier-Gal4* is suppressed in *tor^Æ^* ^P^clones (A–D). In order to identify clones by expression of fluorescent markers, the epidermis was imaged in P9 pupae prior to the onset of pigmentation (A, B). Clones were identified by lack of Ubi-nls-RFP (red), and expression of Rheb was visualized by GFP (green). After live imaging of fluorescently marked clones (dotted lines) in the pupa, the adult fly was recovered to assess the effect of *tor* deletion on pigmentation induced by Rheb-GFP (C, D), the location of the clone was identified by it position relative to the large nuclei of macrochaete bristle cells in the pupa (white arrowheads). Expression of either *raptor^RNAi^* (E), or *s6k1^RNAi^* (F). *UAS-s6k1^TE^*, *pannier-Gal4* flies show mild posterior pigmentation on the thorax (G). The increased pigmentation in the posterior thorax by *pannier-Gal4-*driven overexpression of both *s6k1^TE^* and eIF4E was fully penetrant, but the darkening of the scutellum in this background was not consistently observed in all flies (H). Genotypes of flies: *yw, Ubx-FLP/w; Tor^ΔP^ FRT40A/Ubi-mRFP.nls FRT40A; pannier-Gal4, UAS-Rheb-GFP/+* (A–D). *Y/w, UAS-dicer2; UAS-Rheb/+*; *pannier-Gal4/UAS-raptor^RNAi^* (E), *Y/w, UAS-dicer2; UAS-Rheb/UAS-s6k1^RNAi^*; *pannier-Gal4/+*(F), *Y/w, UAS-dicer2; +/UAS-s6k1^TE^*; *pannier-Gal4/+* (G), *Y/w, UAS-dicer2; +/UAS-s6k1^TE^*; *pannier-Gal4/UAS-eIF4E* raised at 29°C (H).

The TORC1 complex, which contains TOR kinase, is the primary target of Rheb in promoting cell growth (Fig. 1A). We found that Rheb could not drive increased pigmentation in *tor* mutant cells (Fig. 2A–D). However, Tor kinase is a component of two complexes, TORC1 and TORC2. TORC1 is a primary target of Rheb activation and Raptor is the TORC1-specific subunit of the complex that mediates the interaction between TORC1 and its effectors [16]. In order to specifically target TORC1 we crossed *pannier-Gal4*, and *pannier-Gal4*, UAS-Rheb flies to two independent UAS-*raptorRNAi* lines from the TRiP *Drosophila* RNAi collection (TRiP.JF01087 and TRiP.JF01088 [17]). Consistent with TORC1’s role in cell growth, knockdown of Raptor by expression of either UAS-*raptorRNAi* line with *pannier-Gal4* reduced mechanosensory bristle size along a central dorsal stripe on the thorax. *raptor* knockdown also completely suppressed Rheb-induced pigmentation on the thorax and caused diminished pigmentation along the dorsal region of abdominal segments in both the control and Rheb overexpressing flies (Fig. 2E and Fig. S1E–G). These observations lead us to conclude that Rheb-induced pigmentation is TORC1-dependent, but we cannot exclude the possibility that TORC2 may also play some role, since it is unclear whether expression *rictorRNAi*, which failed to suppress either Rheb-induced bristle growth or pigmentation in the thorax, completely abolished TORC2 activity in these flies (Fig. S1H).

TORC1 promotes protein synthesis by phosphorylation of two primary targets: S6 kinase 1 (S6K1) and eIF4E-binding protein (4E-BP). To assess the role of *s6k1* function in both wildtype and Rheb induced pigmentation, we knocked down *s6k1* by RNAi in the thorax with *pannier-*Gal4. *s6k1^RNAi^* stunts mechanosensory bristle growth in both wildtype and Rheb overexpressing flies, but does not suppress melanization in wildtype flies. However, *s6k1^RNAi^* potently suppresses cuticular pigmentation in Rheb overexpressing flies (Fig. 2F). To assess whether S6K1 activity was sufficient to drive increased pigmentation on the thorax, we crossed *pannier-Gal4* to UAS transgenes encoding S6 kinase mutants that mimic an activating phosphorylation (S6K1^TE^) [18]. This activated form of S6K1 markedly enhanced Rheb-dependent pigmentation (Fig. S1I, J). Furthermore, overexpression of the S6K1^TE^ or S6K1^STDETE^ mutant (both which possesses the T_398_ to E amino acid substitution in the linker domain) results in a mild increased pigmentation phenotype on the thorax when pupae are grown at 29°C (Fig. 2G). We hypothesized that since TORC1 activation promotes both S6K1 activity and releases repression on eIF4E, that activation of S6K1 alone was perhaps not sufficient to fully recapitulate the pigmentation phenotype caused by Rheb. We therefore asked whether combined expression of S6K1^TE^ and eIF4E could yield a robust increase in pigmentation on the thorax. Indeed, we find that while overexpression of eIF4E alone has no effect, eIF4E overexpression enhanced the increased pigmentation phenotype resulting from S6K1^TE^ overexpression at 29°C (Fig. 2H). Due to severe distortion of thorax morphology, we were unable to assess whether overexpression of 4E-BP, which acts an inhibitor of eIF4E, could suppress Rheb-induced pigmentation. Taken together, our findings lead us to conclude that Rheb-induced pigmentation on the thorax requires TORC1 complex components Raptor and TOR, and the combined hyperactivity of S6K1 and eIF4E are sufficient to drive darkening of the cuticle.

### Rheb Regulates Catecholamine Biosynthesis in the Thoracic Epidermis

**Figure 3:**
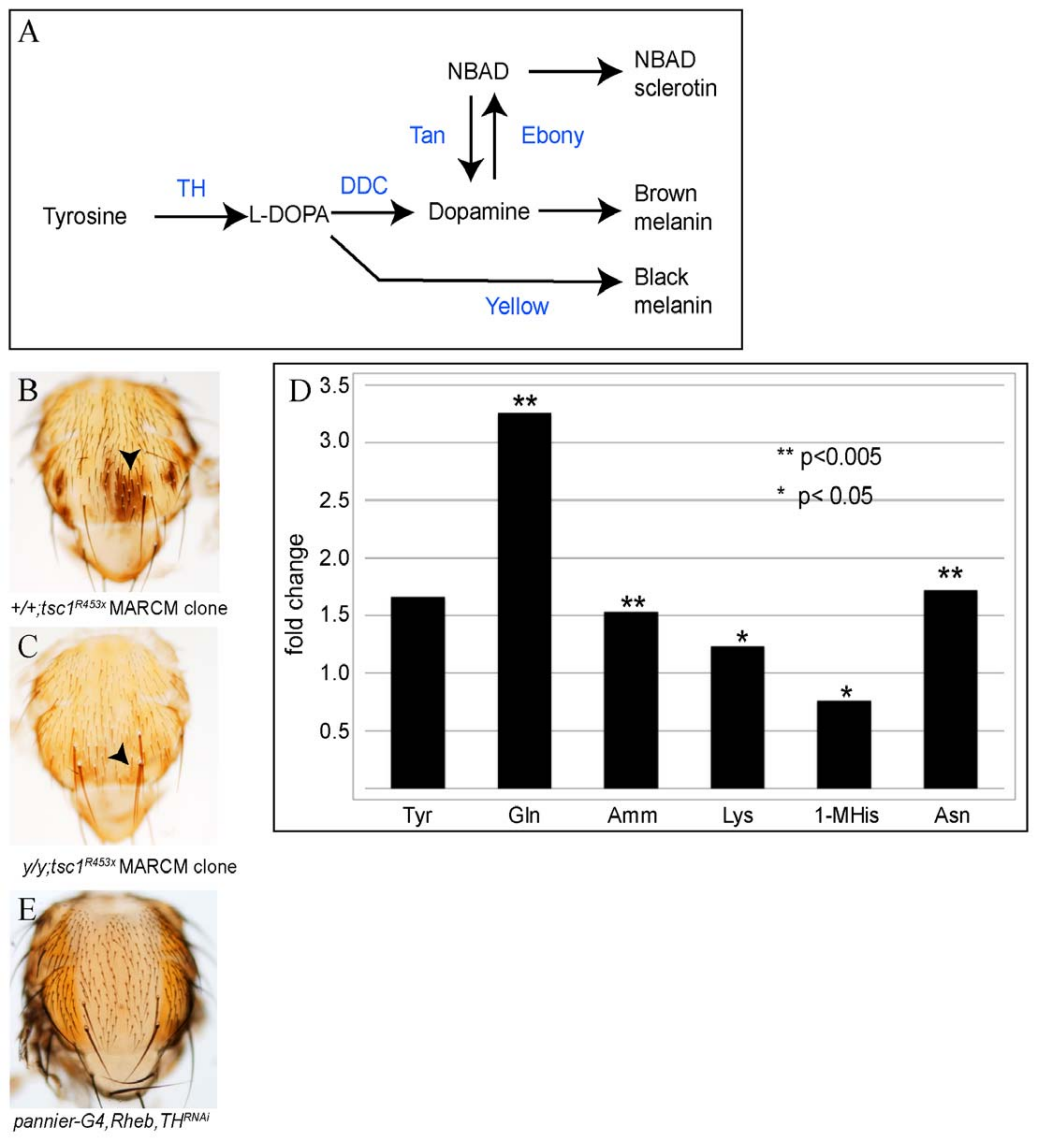
TSC1/2 pathway regulates amino acid levels and function upstream of the catecholamine pathway. The *Drosophila* melanin biosynthesis pathway (modified from (Wittkopp, True and Carroll, 2002) enzymes in blue, substrates in black; phenol oxidases, aaNAT and NADA sclerotin have been excluded) (A). Pigmentation in MARCM clones of *tsc1^ R453x^* (B) is partially suppressed in a *yellow* background (C, arrowheads indicate clone regions in both B and C). Amino acid and metabolite analysis of heads collected from *UAS-Rheb/TM3, Sb* and *elav-Gal4/UAS-Rheb* flies, show statistically significant increases in glutamine, ammonia, lysine, 1-methylhistidine, and asparagine under conditions of neuronal Rheb-overexpression (Student’s T-test-*, D). *UAS-TH^RNAi^* markedly suppressed the *UAS-Rheb*, *pannier-Gal4* pigmentation phenotype (E). Genotypes of flies: *w/yw,Ubx-flp; scabrous-Gal4,UAS-Pon-GFP,UAS-Tau-GFP/+;FRT82B, tsc1^R453x^/FRT82B tub-Gal80* (B), y*w/yw,Ubx-flp; scabrous-Gal4,UAS-Pon-GFP, UAS-Tau-GFP/+; FRT82B, tsc1^R453^/FRT82B tub-Gal80* (C), *Y/w*; UAS-Rheb/TM3, Sb and *Y/w*; UAS-Rheb/*elav-Gal4* (D), *Y/w, UAS-dicer2; UAS-Rheb/+*; *pannier-Gal4/UAS-TH^RNAi^* (E).

Pigmentation in *Drosophila* is based on the synthesis of melanin. Two forms of melanin, brown and black, are synthesized extracellularly from two secreted catecholamine precursors, Dopamine and L-DOPA, respectively. The genes encoding the enzymes directly responsible for melanin synthesis, Tyrosine hydroxylase, DOPA Decarboxylase and Yellow, are induced about 48 hours prior to the emergence of the adult fly [15], [19], and mRNA levels of these enzymes are sustained in through eclosion of the adult fly. After eclosion, the fly cuticle darkens and hardens due to the activation of a neuropeptide cascade [15]. The first step in *Drosophila* melanin biosynthesis is the conversion of tyrosine to L-DOPA by the activity of the Tyrosine Hydroxylase enzyme (TH, encoded by the *pale* gene) (Fig. 3A). DOPA acts as a substrate for Dopa Decarboxylase (DDC) and Yellow, enzymes that produce dopamine and black melanin, respectively. Dopamine is converted to brown melanin through phenol oxidase activity [20]. Ebony, an N-β-alanyl dopamine (NBAD) synthetase enzyme, also controls pigmentation levels in the cuticle by diverting dopamine away from melanin and toward NBAD sclerotin synthesis (Fig. 3A)[19]–[21]. We therefore conducted several genetic experiments to determine whether manipulation of the pigment pathway alters the Rheb-dependent pigmentation. First, we found that Rheb-induced pigmentation is modulated by Ebony levels (Fig. S2A–E). Second, increased pigmentation in *tsc1* mutant clones is partially suppressed in a *yellow* mutant background: the cuticle within the *tsc1* clone exhibits a brown hue, while the surrounding tissue is a lighter yellow color, suggesting that black melanin pigment is suppressed in the *yellow* mutant, while L-DOPA-dependent brown melanin persists (Fig. 3A–C).

These observations demonstrate that Rheb is likely acting upstream of Ddc by potentially increasing either the levels of the substrate, tyrosine, or expression of the rate-limiting enzyme, TH, in the melanin pathway. We therefore tested whether cells with high Rheb activity contain higher levels of tyrosine. We measured free amino acid levels from heads of Rheb overexpressing flies (using the neuronal driver *elav-Gal4*) and found significantly higher levels of several amino acids and their metabolites, however, although tyrosine levels showed a trend towards increase in Rheb overexpressing samples, the change did not reach statistical signficance (Fig. 3D). We hypothesized that TH is required for the Rheb-induced pigmentation phenotype. Indeed, in both wildtype and Rheb overexpressing flies, *TH^RNAi^* potently suppressed cuticular pigmentation, resulting in a hypo-pigmented region of cuticle along the dorsal thorax coincident with the *pannier-Gal4* expression domain (Fig. 3E). These findings point to TSC1/2 and Rheb in regulating melanization upstream of the catecholamine biosynthesis pathway in the epidermis.

### Rheb Activity Regulates Tyrosine Hydroxylase

**Figure 4:**
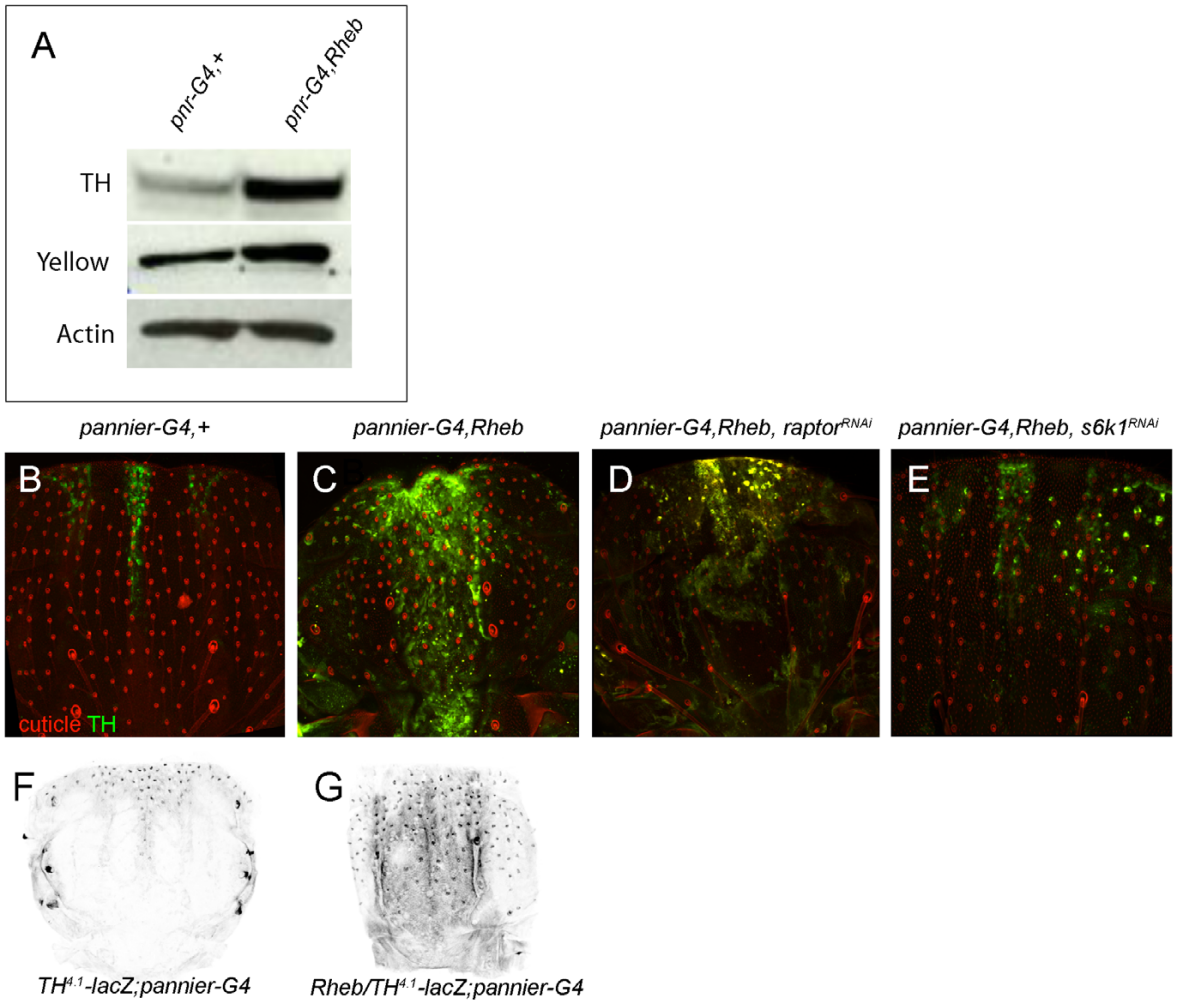
Rheb activity drives increased TH levels in pupal epidermal cells. Western blot analysis reveals a robust increase in levels of TH protein, and more modest increase of Yellow protein, in Rheb overexpressing thoraces compared to *pannier-Gal4* (*pnr-G4*) line alone (A). TH protein is expressed in a subset of anterior epidermal cells prior to the onset of pigmentation in the P10 stage pupal thorax (B). *UAS-Rheb, pannier-Gal4* pupa showing increased numbers of TH protein expressing cells along the central dorsal region of the thorax (C), which is suppressed by either *raptor^RNAi^* (D), or *s6k1^RNAi^* (E). Overexpression of Rheb by *pannier-Gal4* expands the expression of the *TH^4.1^-LacZ* reporter, as shown by β-gal labeling (gray, F, G). Genotypes of flies: *Y/w, UAS-dicer2; pannier-Gal4/+* (A, B, G), *Y/w, UAS-dicer2; UAS-Rheb/+*; *pannier-Gal4/+* (A, C, G), *Y/w, UAS-dicer2; UAS-Rheb/+*; *pannier-Gal4/UAS-raptor^RNAi^*(D), *Y/w, UAS-dicer2; UAS-Rheb/UAS-s6k1^RNAi^*; *pannier-Gal4/+*(E), *Y/w, UAS-dicer2;+/TH^4.1^-LacZ, pannier-Gal4/+* and *Y/w, UAS-dicer2; UAS-Rheb/TH^4.1^-LacZ*; *pannier-Gal4/+*(F).

TH, DDC, Ebony and Yellow protein levels increase during late pupal stages coinciding with the onset of pigmentation [15], [19], [22]. We therefore asked whether TH protein levels were altered in Rheb overexpressing pupae. In contrast to the modest rise in Yellow, we found that TH protein levels were strongly increased in staged thoraces of Rheb overexpressing pupae at the onset of pigmentation (Fig. 4A). In order to visualize the pattern of TH protein expression, we performed immunohistochemical labeling of TH in isolated thoraces just prior to the onset of pigmentation, in both wildtype and Rheb overexpressing flies. Pupal bristle pigmentation is induced in an anterior to posterior wave at stage p10, and TH expression is likewise induced in a small subset of epidermal and mechanosensory bristle cells at the anterior region in control pupae (*pannier*-Gal4). On the other hand, at this same developmental stage in Rheb overexpressing pupae, the TH expression domain extends to the posterior region of the thorax and TH is expressed in many more cells (Fig. 4B–C). Consistent with our previous observations of Rheb induced pigmentation, expansion of the TH expression domain in the Rheb overexpressing flies was suppressed by expression of either *raptor^RNAi^* or *s6k1^RNAi^* (Fig. 4B–E). Elevated TH protein levels could be due to increased transcription, translation, or protein stability. We asked whether Rheb overexpression could promote expression of a lacZ reporter construct, that recapitulates the expression pattern of endogenous TH [23]. In both wildtype and Rheb-overexpressing pupae, the TH lacZ reporter expression pattern was similar to that observed with the TH antibody (Fig. 4F, G), which suggests that Rheb controls TH through either transcription or translation, but is not dependent on the TH coding sequence. Despite the strong increase in TH protein in isolated thoraces from *pannier-Gal4*, UAS-Rheb pupae, we did not observe significant increase in *tyrosine hydroxylase* RNA levels by rtPCR, while Rheb levels showed a three fold increase (Fig. S2F), Taken together, our findings indicate that high Rheb activity increases TH expression in epidermal and mechanosensory cells in the pupal thorax.

## Discussion

Our study demonstrates that high Rheb activity in epidermal cells of the fly results in increased levels of melanin synthesis and pigmentation during pupal development. Rheb-induced hyper-pigmentation is TORC1-dependent and appears to be due to increased levels of tyrosine hydroxylase (TH) protein, the rate-limiting enzyme in catecholamine biosynthesis. Adult *Drosophila* cuticular pigmentation occurs in two steps: in the first, initiated during late pupal stages, melanization genes such as TH, DDC, Yellow and Ebony are expressed in the epidermis and external cells of the mechanosensory organs in order to commence pigmentation, and in the second, after eclosion, a burst of pigmentation activity occurs that is controlled by a neuropeptide cascade, which is required for cuticular tanning and hardening of the adult cuticle [15], [19]. We show that Rheb promotes premature pigmentation of the mechanosensory bristles during the pupal stage, and also drives darkening of the posterior cuticle of the thorax after eclosion. It is unclear why this increased pigmentation is biased to the posterior region, known as the trident, but key pigmentation enzymes such as Yellow and Ebony, are expressed at different levels in this part of thorax, suggesting that this region may be more sensitive to changes in catecholamine levels.

While our data indicates that Rheb activity increases TH protein levels, it is unclear whether this is through a transcriptional or post-transcriptional regulation. Previous studies have indicted that TH is translationally repressed during pupal eclosion, but the mechanism of this repression is not well understood [15]. TORC1, through the combined activities of both S6K and eIF4E activities, promotes recruitment of the initiation factor complex to mature mRNAs thereby increasing protein synthesis [24]–[26]. Although we saw increases in both protein levels of Yellow and TH when Rheb was overexpressed, TH levels were markedly higher, while its mRNA levels did not show an increase. These finding point to the possibility that TH translation may be limited by TORC1 activity in wildtype cells. High TORC1 activity promotes the unwinding of mRNAs with long and structured 5′ UTRs by the helicase subunit of the initiation complex eIF4A [27]. The TH 5′UTR is longer and predicted to be more structured than the *yellow* 5′ UTR and knockdown of eIF4A blocks Rheb-induced hyperpigmentation (Fig. S2G, H). High Rheb levels could therefore increase translation rates of TH without increasing the level of TH mRNA. We cannot exclude however that activation of Rheb may, directly or indirectly, also increase levels of transcription or stability of the TH mRNA that was not detected in our rtPCR experiments. The fact that we observe premature pigmentation in *tsc1* clones is reminiscent of the precocious differentiation of *tsc* mutant photoreceptors in the fly eye [7], suggesting that TORC1 activity may be a limiting factor in regulating the timing of differentiation in a variety of developmental contexts.

In addition to growth of hamartomous tumors in the skin, kidney, lungs, heart and brain, human patients with TSC exhibit non-tumorous skin lesions, such as hypopigmented macules and shagreen patches, and neurological symptoms including developmental delay, seizure and autism [6]. In *Drosophila*, the catecholamine biosynthetic pathway is used in the adult cuticle to produce intermediates for melanin and sclerotin synthesis, as well as a source of dopamine in dopaminergic neurons in the brain. Although melanogenesis in vertebrates relies on tyrosinase to convert tyrosine to dopa, the neural dopamine pathway is highly conserved between flies and vertebrates [28]. Our findings suggest that increased levels of rate-limiting enzyme, TH, may result in dysregulation of catecholamine biosynthesis. Such a mechanism could contribute to the skin lesions, tumors and neurological manifestations observed in human patients, for example by increasing protein levels of oncogenes, metabolic enzymes, neurotransmitters or neuromodulators that are normally under tight translational control [29], [30]. Future in vivo studies focusing on cell type-specific TORC1-dependent protein level changes in mutant tissues will provide further insights into the mechanisms driving tumor growth and neural dysfunction in TSC disease.

## Materials and Methods

### *Drosophila* Genetics, Live Imaging, and Immunohistochemistry

Genotypes of *Drosophila* strains used in this study are provided in the supplementary material. Unless otherwise noted, *Drosophila* stocks and crosses were maintained at 22°C on standard media. For mounting adult cuticles, flies were collected, stored and dissected in 80% isopropanol, then cleared and mounted in Hoyer’s media. The Rheb-GFP transgenes were generated by fusing the coding sequence of Rheb to GFP at the carboxy-terminus using the gateway system (Invitrogen), which were then injected into *Drosophila* embryos intergrated into the genome through P-element insertion. For visualization of GFP marked *tsc1* and *tsc2* clones in pupae, pupa were removed from the pupal case at stages P10 and mounted as described in [31], and imaged on a Nikon C1 Confocal microscope as described in [32]. Autofluorescence of the stage P9-10 pupal cuticle is revealed by excitation at 488 nm and acquisition of emission at 590 nm/50 filterset. For immunohistochemical analysis, Stage P10 pupae were dissected and fixed as described in [32]. The TH lacZ construct contains a 4 kb fragment of genomic sequence upstream of the TH coding region which includes 361 base pairs (of the 451 bp total) TH 5′UTR and replaces the TH coding region with lacZ. The rabbit anti-*Drosophila* Tyrosine Hydroxylase antibody (a generous gift from W. Neckameyer) was used at 1∶500, and mouse –anti β-gal was used at 1∶1000.

### Amino Acid Analysis

In order to analyze amino acid levels in wildtype and Rheb overexpressing cells in flies, we crossed homozygous UAS-Rheb flies to *elav-*Gal4/TM3, Sb and collected 3 sets of 200 flies each of the UAS-Rheb/TM3 and UAS-Rheb/*elav-*Gal4 genotypes. Flies were frozen in liquid nitrogen and stored at −80°C, then manually decapitated. Each set of heads was homogenized in equal volume (400 µl) of 2.5% sulfosalicylic acid, followed by centrifugation at 10,000 rpm for 15 minutes. All steps were done at 4°C. The clear supernatant was then analyzed using the Biochrom 30 amino acid analyzer (Biochrom, Cambridge, UK).

### Western Blots and RT-PCR

Stage P10 pupae were collected and the dorsal thoraces were isolated by manual dissection. For real-time PCR twelve thoraces were collected for RNA extraction using the RNAeasy kit (Qiagen). Probesets used for RT-PCR: TH (TTGAGGAGGATGTTGAGTTTGAGA and CTCGGTGAGACCGTAATCGTT), Rheb (TGAGGTGGTGAAGATCATATACGAA and GCCAGCTTCTTGCCTTCCT) were run using Taqman/and spt4 control (CTCGTGGTACTCCTGCCATTTCTG and TCCACGATTCTTCATGTCACGTA) using cybergreen. Rheb and TH RNA levels were normalized to Spt4 levels in both control and Rheb overexpressing samples. For Western blots fifteen thoraces were collected, homogenized in RIPA buffer, run on a gel and protein transferred to a nitrocellulose membrane. Antibodies used for Western blot were Rabbit anti-Yellow (1∶1000, generous gift from S. Carroll), rabbit anti-Tyrosine hydroxylase (1∶1000, W. Neckameyer), and mouse anti-actin (Sigma).

## Supporting Information

Figure S1 **Rheb overexpression increases pigmentation on the thorax and abdomen.** Male *pannier-Gal4* abdomen, showing the narrow dorsal pigment stripe in segments A3 and A4 (A). Rheb overexpression expands the dorsal pigment stripe (B). The Rheb^AV4^ allele crossed to *pannier-Gal4* shows a pigment patch on the thorax (C), and TSC2^RNAi^ knockdown expands the dorsal pigment stripe (D). Raptor knockdown (*raptor^RNAi^* lines TRiP.JF01087 and TRiP.JF01088 (Kockel, Kerr, Melnick, *et al*, 2010)) suppressed Rheb-induced expansion of the dorsal pigment stripe on the male abdomen (E–F). *rictor^RNAi^* (TRiP.JF01370) does not suppress Rheb-induced pigmentation on the thorax (H). Overexpression of either S6K1^TE^ or S6K1^STDETE^ enhances the thoracic Rheb-induced pigmentation (I, J).
(TIF)

Figure S2 **Rheb induced Pigmentation is modulated by** ***ebony*** **.** Compared to Rheb-overexpressing controls (A), *ebony* heterozygous mutant flies overexpressing Rheb exhibit a more pronounced posterior pigment patch on the thorax (B). Overexpression of Ebony suppresses the Rheb-induced pigmentation on the thorax (C), while pigmentation in *pannier-Gal4, ebony^RNAi^* (D) is enhanced by Rheb overexpression (E). Fold change of Rheb and TH transcripts between *UAS-Rheb, pannier-Gal4,* and *pannier-Gal4* thoraces. Rheb shows a 3.5 fold change, but no detectable change of TH (Wilcoxon test -*, F). Knockdown of the helicase eIF4A (using the TRiP line HMS00927) suppresses the bristle growth and increased pigmentation driven by Rheb in the pupal thorax (G). TH and Yellow 5′UTRs. Predicted secondary structure and probability of base pairing of the *tyrosine hydroxylase* and *yellow* 5′UTR using the RNAFold algorithm (bp =  base pairs, minimum free energy calculation is shown in blue text, H).
(TIF)
