# Supplementary figures and images for: The TSC1/2 Complex Controls Drosophila Pigmentation through TORC1-Dependent Regulation of Catecholamine Biosynthesis
Source: PLoS One. 2012 Nov 7;7(11):e48720. doi: 10.1371/journal.pone.0048720 (PMC3492411; doi:10.1371/journal.pone.0048720)

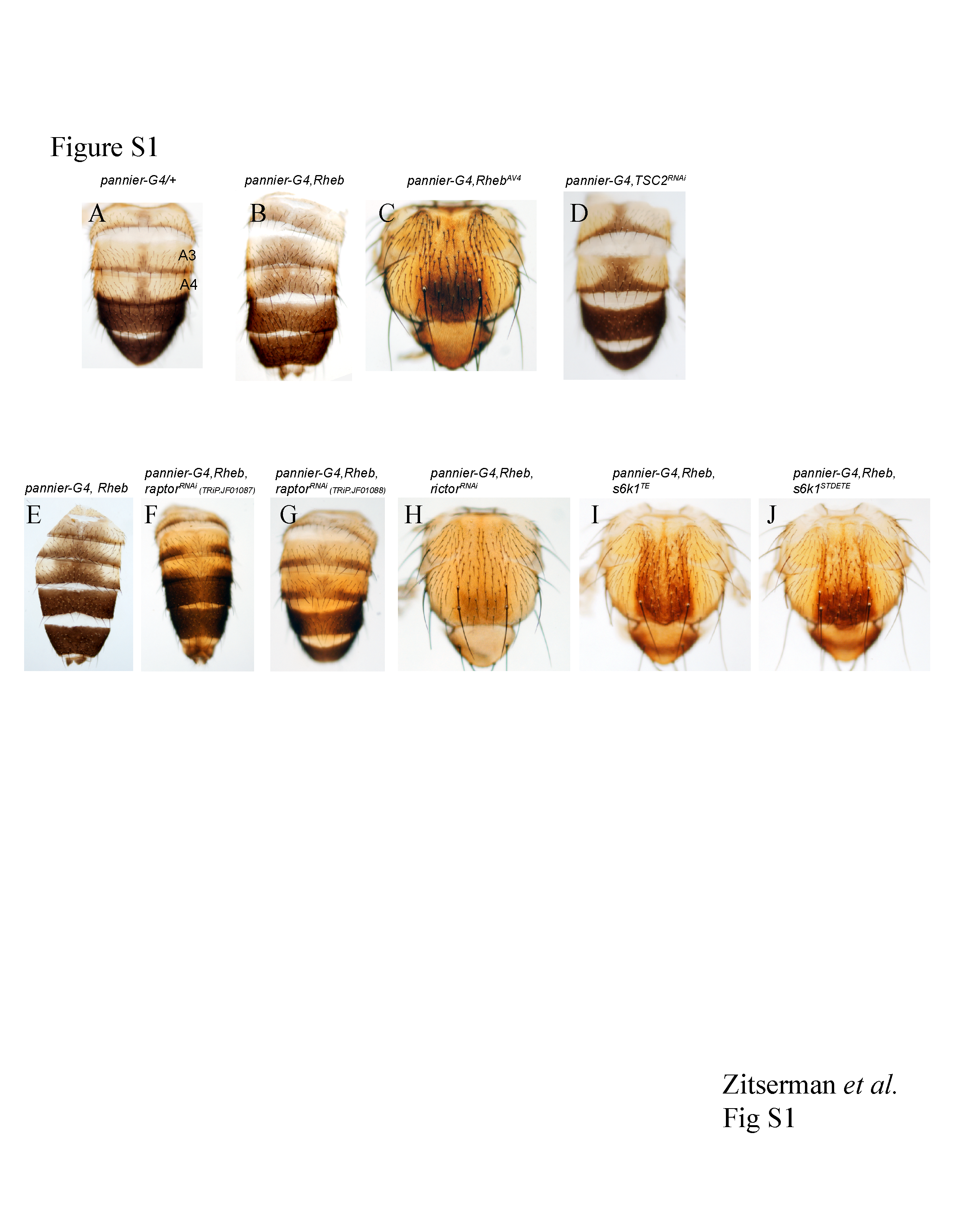

Supplement: Figure S1 — Rheb overexpression increases pigmentation on the thorax and abdomen. Male pannier-Gal4 abdomen, showing the narrow dorsal pigment stripe in segments A3 and A4 (A). Rheb overexpression expands the dorsal pigment stripe (B). The RhebAV4 allele crossed to pannier-Gal4 shows a pigment patch on the thorax (C), and TSC2RNAi knockdown expands the dorsal pigment stripe (D). Raptor knockdown (raptorRNAi lines TRiP.JF01087 and TRiP.JF01088 (Kockel, Kerr, Melnick, et al, 2010)) suppressed Rheb-induced expansion of the dorsal pigment stripe on the male abdomen (E–F). rictorRNAi (TRiP.JF01370) does not suppress Rheb-induced pigmentation on the thorax (H). Overexpression of either S6K1TE or S6K1STDETE enhances the thoracic Rheb-induced pigmentation (I, J). (TIF) [file pone.0048720.s001.tif]

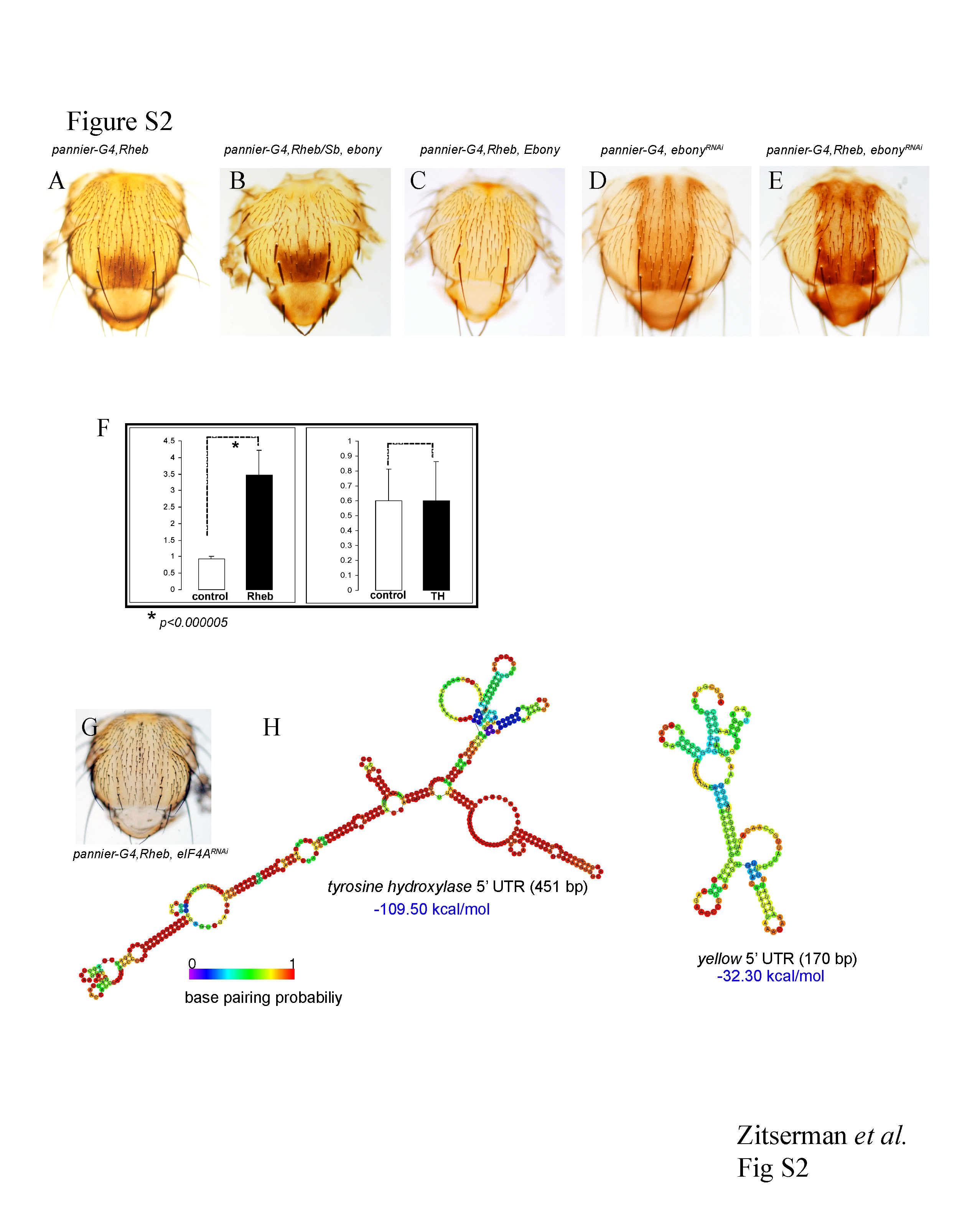

Supplement: Figure S2 — Rheb induced Pigmentation is modulated by ebony . Compared to Rheb-overexpressing controls (A), ebony heterozygous mutant flies overexpressing Rheb exhibit a more pronounced posterior pigment patch on the thorax (B). Overexpression of Ebony suppresses the Rheb-induced pigmentation on the thorax (C), while pigmentation in pannier-Gal4, ebonyRNAi (D) is enhanced by Rheb overexpression (E). Fold change of Rheb and TH transcripts between UAS-Rheb, pannier-Gal4, and pannier-Gal4 thoraces. Rheb shows a 3.5 fold change, but no detectable change of TH (Wilcoxon test -*, F). Knockdown of the helicase eIF4A (using the TRiP line HMS00927) suppresses the bristle growth and increased pigmentation driven by Rheb in the pupal thorax (G). TH and Yellow 5′UTRs. Predicted secondary structure and probability of base pairing of the tyrosine hydroxylase and yellow 5′UTR using the RNAFold algorithm (bp = base pairs, minimum free energy calculation is shown in blue text, H). (TIF) [file pone.0048720.s002.tif]
